# Potential Distribution of *Anoplophora horsfieldii Hope* in China Based on MaxEnt and Its Response to Climate Change

**DOI:** 10.3390/insects16050484

**Published:** 2025-05-02

**Authors:** Dan Yong, Danping Xu, Xinqi Deng, Zhipeng He, Zhihang Zhuo

**Affiliations:** College of Life Science, China West Normal University, Nanchong 637002, China; yongdan1993@foxmail.com (D.Y.); danpingxu@foxmail.com (D.X.); deng.xinqi@foxmail.com (X.D.); zhipeng_hh@foxmail.com (Z.H.)

**Keywords:** *Anoplophora horsfieldii Hope*, MaxEnt, species distribution models, climatic suitability prediction, climate change, China

## Abstract

*Anoplophora horsfieldii Hope*, a potential pest of the Cerambycidae family, is widely distributed throughout China, where it can cause damage to various living tree species. It has emerged as a critical invasive organism threatening China’s agricultural and forestry production as well as ecological security. This study comprehensively analyzed the key environmental factors influencing the geographical distribution of *A. horsfieldii* and its spatiotemporal dynamics by integrating multi-source environmental data and employing ecological niche modeling. Model validation demonstrated high reliability and accuracy of our predictions, with an area under the receiver operating characteristic curve (AUC) value of 0.933, Kappa coefficient of 0.704, and true skill statistic (TSS) reaching 0.960. Our analysis identified four dominant environmental factors governing the distribution of *A. horsfieldii*: mean diurnal range (Bio2), temperature annual range (Bio7), precipitation of driest quarter (Bio17), and precipitation of coldest quarter (Bio19). Under current climatic conditions, the total potential suitable distribution area for *A. horsfieldii* was estimated at 212.394 × 10⁴ km^2^, primarily located in central, southern, eastern, southwestern, and northwestern China. Future projections under three climate scenarios (SSP1-2.6, SSP2-4.5, and SSP5-8.5) suggest significant reductions in highly and moderately suitable habitats, while low-suitability areas may expand into central, eastern, and southwestern regions, with Chongqing, Henan, and Anhui potentially becoming new suitable habitats. Concurrently, the centroid coordinates of suitable habitats exhibited a directional shift toward Guangdong Province, with the overall distribution pattern demonstrating a spatial transition characterized by movement from inland to coastal areas and from higher to lower latitudes. This study provides scientific theoretical support for forestry authorities in controlling the spread of *A. horsfieldii*, while establishing a solid foundation for future ecological conservation and biosecurity strategies. The findings offer both theoretical insights and practical guidance for pest management and ecosystem protection.

## Simple Summary

*Anoplophora horsfieldii* is a potential pest that threatens forestry and ecological security in China. Based on ecological niche modeling, this study found that the distribution of *A. horsfieldii* is primarily influenced by temperature and precipitation. Currently, its potential suitable habitat covers approximately 212.394 × 104 km^2^, mainly concentrated in central China, south China, east China, southwest China, and northwest China. Future climate scenario projections suggest that the areas classified as highly and moderately suitable will undergo a significant reduction, whereas low-suitability areas are expected to expand further into central, eastern, and southwestern China, with regions such as Chongqing, Henan, and Anhui likely to become new suitable habitats. Moreover, the centroid of the suitable habitat is projected to shift toward Guangdong Province, reflecting a spatial migration trend from inland to coastal regions and from higher to lower latitudes. This study provides scientific theoretical support for forestry authorities in controlling the spread of *A. horsfieldii*, while establishing a solid foundation for future ecological conservation and biosecurity strategies. The findings offer both theoretical insights and practical guidance for pest management and ecosystem protection.

## 1. Introduction

*Anoplophora horsfieldii Hope*, a species of woodboring beetle in the family Cerambycidae, is widely distributed in China and the Indochinese Peninsula, including India, Laos, Taiwan, Thailand, and Vietnam [1]. This species exhibits distinct diurnal activity patterns, with peak emergence periods varying geographically—primarily occurring from May to July in Taiwan, while observed between July and August in southeastern China [2]. Recent studies have confirmed that *A. horsfieldii* has become the third species within the Cerambycidae family identified as having invasive characteristics [3]. This species exhibits strong host adaptability, with a wide range of economically important host tree species, including *Camellia oleifera* Abel, 1818; *Camellia sinensis* (Linnaeus) Kuntze, 1887; *Ulmus pumila* Linnaeus, 1753; *Melia azedarach* Linnaeus, 1753; *Quercus glauca* Thunberg, 1784; and *Celtis* spp. [2,4,5,6,7,8]. Its broad host range poses a significant threat to both agricultural and forest ecosystems. For instance, in the Yongyeon Valley of Jeju Island, South Korea, systematic surveys over the past three years recorded 30 adult individuals, all concentrated in this area. Among 32 infested Celtis trees, four exhibited partial or complete dieback due to intensive larval feeding, as indicated by the presence of numerous emergence holes [9]. This pattern of damage not only directly impairs the normal growth and development of trees but also carries the potential for substantial economic losses in the forestry and agriculture sectors. Of particular concern, research indicates that *A. horsfieldii* is capable of establishing populations throughout Jeju Island, with the exception of high-altitude areas on Mount Halla. Moreover, it may further spread to parts of Southeast and East Asia, including southern Korea and Japan [10]. Given its documented impact in current habitats and its rapid adaptability in newly invaded regions, the potential ecological and economic consequences of *A. horsfieldii* warrant urgent attention and proactive management strategies.

Global climate change and the expansion of international trade are intensifying the introduction and spread of harmful pests. With the increasing development of global transportation networks, insect species are rapidly crossing geographic boundaries and expanding into new ecosystems [11,12,13]. While trade facilitates the global spread of insect species, their ability to establish and further spread in new environments still depends on the adaptability of local climate conditions [14]. Climate change, by opening new pathways for spread and altering ecological interactions [15], has greatly accelerated the invasion and expansion of non-native insect species [16]. As climate change continues, the distribution patterns of species will shift, ecological relationships and food chains will be disrupted, and the threat of invasive species will intensify, leading to profound impacts on ecosystem stability and biodiversity conservation [17].

Species distribution models (SDMs) play a critical role in assessing the impacts of climate change on potential insect distributions. The maximum entropy (MaxEnt) model, as an advanced species distribution prediction tool, has been widely applied in pest control and ecological research [18,19,20]. This model estimates potential suitable habitats by integrating known species-occurrence data with environmental variables while minimizing the relative entropy (Kullback–Leibler divergence) between species distribution and environmental background distribution [21]. Even with limited occurrence data, MaxEnt can provide highly accurate predictions, demonstrating both high precision and broad applicability [22]. Due to these advantages, it has become an essential tool for pest distribution prediction. For instance, Yasser Alramadan et al. used MaxEnt to predict habitat suitability for *Anoplophora chinensis* under CMIP6 climate scenarios [23], showing suitable habitat expansions of 5.99–69.33% under SSP1-2.6 and 13.31–45.78% under SSP5-8.5. Additionally, Quancheng Zhang et al. employed MaxEnt to model potential distributions of *Anoplophora glabripennis* and its natural enemies in China [24], revealing northward shifts of suitable areas under climate change, with *A. glabripennis* primarily distributed in parts of Xinjiang, Tibet, and Qinghai.

This study developed a predictive model for the potential suitable habitats of *A. horsfieldii* in China by integrating three representative climate scenarios from the Shared Socioeconomic Pathways (SSPs) under the Coupled Model Intercomparison Project Phase 6 (CMIP6) framework [25]. This scenario selection strategy holds significant scientific value: first, it comprehensively covers the greenhouse gas emission gradient range defined in the IPCC Sixth Assessment Report; second, it systematically evaluates the ecological stress effects on species from gradual to abrupt climate changes; third, it aligns with international research frontiers in climate change ecology. Through multi-scenario coupling analysis, this study systematically reveals for the first time the potential distribution patterns of this species across mainland China. Compared to previous studies on the invasion status of *A. horsfieldii* in South Korea [26], particularly the work by Min-Jung Kim et al. (2025), which systematically analyzed the species’ climatic adaptability and dispersal mechanisms on Jeju Island using species distribution models (SDMs) [10], the present study is the first to apply the MaxEnt model to predict its potential distribution across the entire territory of China, thereby filling a research gap in the application of this method within the country. Given the potential threats posed by *A. horsfieldii* to ecological environments, as well as agricultural and forestry economies, in-depth research on its suitable habitats, distribution patterns, and ecological characteristics is of paramount importance for developing scientifically sound and effective control strategies. This study not only provides a theoretical foundation for the ecological management of *A. horsfieldii* but also establishes a robust scientific basis for future ecological conservation and biosecurity practices.

## 2. Materials and Methods

### 2.1. Collection and Selection of A. horsfieldii Distribution Data

**Figure 1:**
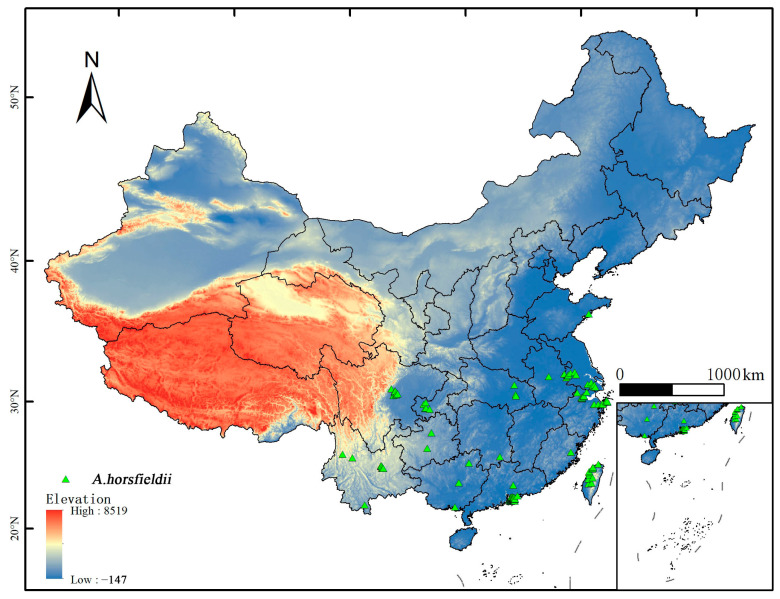
The occurrence sites of *A. horsfieldii* in China.

The distribution data of *A. horsfieldii* were primarily obtained from the Global Biodiversity Information Facility (GBIF, http://www.gbif.org; accessed 24 March 2025) and supplementary literature. A total of 275 occurrence records were compiled, comprising 273 GBIF records (based on human observations) and 2 literature-derived records. To address potential model overfitting caused by spatial autocorrelation and sampling biases in the raw distribution data, we implemented spatial filtering using ENMTools. This data-thinning process retained only one unique occurrence record per 10 × 10 km grid cell, resulting in a refined dataset of 144 spatially independent records (Figure 1) that served as the foundational data for subsequent model calibration and validation.

### 2.2. Acquisition and Processing of Environmental Variables

Environmental data include 19 climate bioclimatic factors (bio1–19) and 1 geographic factor (elevation). The 19 global bioclimatic variables in this study were obtained from the WorldClim 2.1 database (website: www.worldclim.org) with a resolution of 5 arc minutes, covering the time period from 1970 to 2000. Elevation data were extracted from the Digital Elevation Model (DEM) provided by the Geospatial Data Cloud (http://www.gscloud.cn/), with the resolution consistent with that of the climate factors. For future climate conditions (i.e., 2041–2060 and 2081–2100), three scenarios from the Beijing Climate Center Climate System Model (BCC-CSM2-MR) under the sixth phase of the Coupled Model Intercomparison Project (CMIP6) were used for prediction: low-emission scenario (SSP1-2.6), medium-emission scenario (SSP2-4.5), and high-emission scenario (SSP5-8.5). To avoid multicollinearity among the variables and minimize interference with the prediction results, environmental factors were selected before modeling to ensure the maximum influence on the species distribution. A Pearson correlation analysis was conducted for all variables, and when the correlation coefficient (|r|) between two variables exceeded 0.8, important climate variables were chosen based on their contribution to the potential distribution of *A. horsfieldii* in the initial MaxEnt model construction. Variables with higher maximum entropy gain were retained for subsequent MaxEnt modeling analysis (Table 1).

### 2.3. The Optimization and Construction of MaxEnt Model Parameters

**Figure 2:**
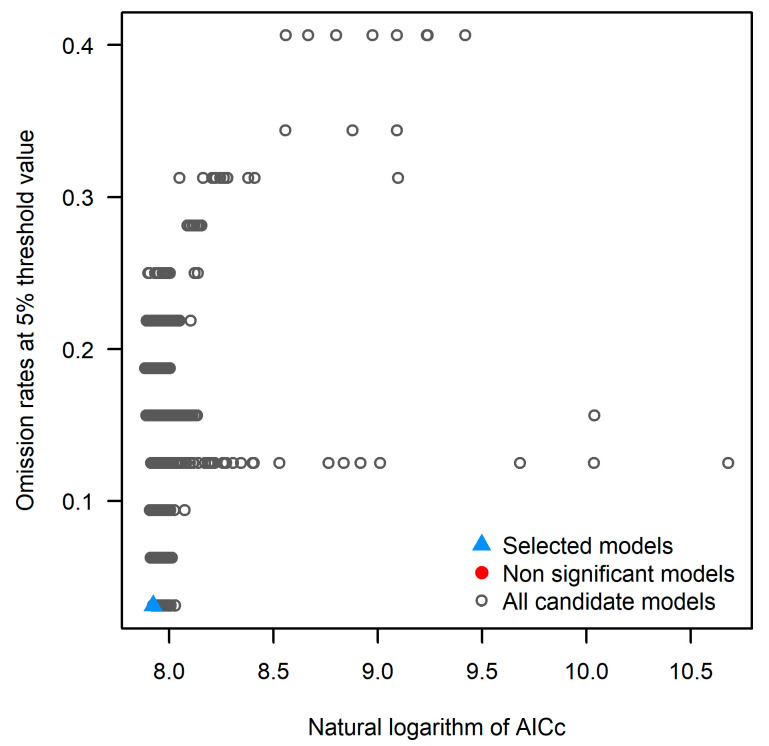
Distribution of AICc and omission rates for candidate models with optimal model.

This study conducted a systematic analysis of the potential suitable habitats for *A. horsfieldii* using the Kuenm platform (based on the MaxEnt algorithm) integrated with the ArcGIS 10.8 geographic information system [27]. Through a comprehensive model calibration process, we evaluated 1240 candidate models with parameter combinations including 40 regularization multipliers (0.1–4.0, increment 0.1), 31 feature classes (containing individual and combined L/Q/P/T/H features), and 1 environmental variable set (set_1). Model evaluation employed three key criteria: statistical significance (Partial ROC, *p* < 0.05), omission rate (OR ≤ 5% threshold), and small-sample-size corrected AICc (Akaike Information Criterion). After rigorous screening, all candidate models demonstrated statistical significance, with 180 meeting the omission-rate criterion. The optimal model M_2_F_qt_set_1 (“M2” indicates a regularization multiplier (RM) of 2.0; “Fqt” refers to the feature combination of quadratic (Q) and threshold (T); “set_1” denotes the first set of environmental variable combinations used) exhibited exceptional performance metrics: AUC ratio (defined as the ratio between the model’s AUC and the expected AUC of a random classifier, which is 0.5) of 1.819, omission rate of 0.031 (5% threshold), AICc value of 2761.581 (ΔAICc = 0, weight W_AICc = 1), and 29 parameters, demonstrating optimal balance on the Pareto front while satisfying all criteria of statistical significance, low omission rate (3.1%), and minimal AICc value (Figure 2). All environmental variables were preprocessed in ArcGIS 10.8 and imported in ASCII format. During model computation, 75% of occurrence data served as training samples while the remaining 25% were used for validation, with 10,000 maximum iterations and 10 replicate runs using the Subsample method to ensure model robustness. Finally, we assessed the constraining effects of climatic variables on the species’ distribution through calculations of percent contribution and permutation importance values.

The accuracy of the simulation results was evaluated using the receiver operating characteristic (ROC) curve, where the area under the curve (AUC) represents the area under the ROC curve. The AUC value ranges from 0 to 1, with higher values indicating better model-prediction accuracy and reliability [28]. The evaluation of the AUC value is divided into five levels: when 0.5 ≤ AUC < 0.6, it indicates model-prediction failure; 0.6 ≤ AUC < 0.7 indicates poor prediction accuracy; 0.7 ≤ AUC < 0.8 indicates average prediction accuracy; 0.8 ≤ AUC < 0.9 indicates good prediction accuracy; and 0.9 ≤ AUC < 1 indicates excellent prediction accuracy. In practice, an AUC value greater than 0.8 typically indicates high accuracy of the simulation results [29]. The simulation results are output using the Logistic method and saved in .asc format on the desktop, with other parameters set to the default values of the MaxEnt 3.4.4 software. The MaxEnt 3.4.4 simulation results are then visualized and divided into climatic suitability categories for *A. horsfieldii* using ArcGIS 10.8. Based on the simulated suitability levels and the collected site distribution data, the suitable habitat was classified into four levels using the following thresholds: unsuitable area (0–0.1), low-suitability area (0.1–0.3), medium-suitability area (0.3–0.5), and high-suitability area (0.5–1.0). The Reclassify tool in ArcGIS 10.8 was then used to calculate and analyze the area corresponding to each suitability level.

### 2.4. Centroid Migration Analysis

This study focuses on the suitable habitat of *A. horsfieldii*, aiming to explore the spatial variation trends of its suitable habitat centroid under different climate scenarios by analyzing the changes in the centroid of its suitable habitat during the present and future periods. By using the SDM Toolbox v2.4 in ArcGIS, we generated vector files of centroid changes in suitable habitats across adjacent time periods and further analyzed the migration patterns of the *A. horsfieldii* habitat centroid in response to climate change.

## 3. Results

### 3.1. MaxEnt Model Accuracy Assessment and Identification of Key Environmental Variables

**Figure 3:**
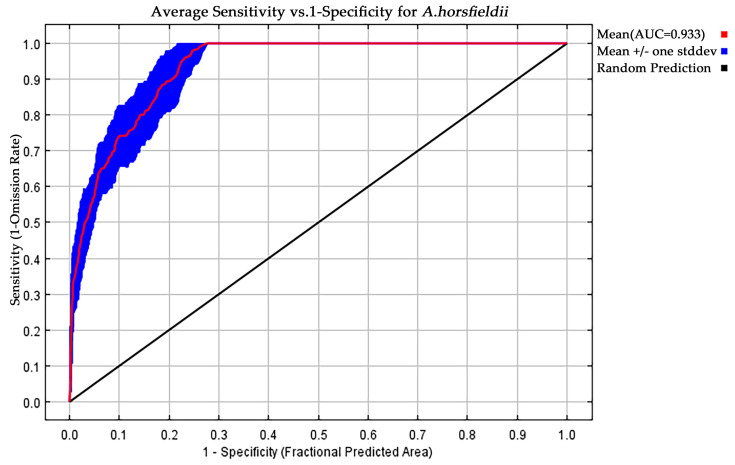
The receiver operating characteristic (ROC) curve.

**Figure 4:**
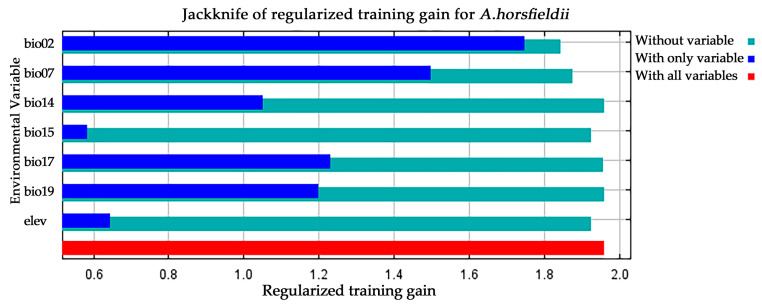
Jackknife test to examine training gain results of environmental variables.

Accuracy analysis of the MaxEnt model and the selection of key environmental variables are crucial steps in evaluating its predictive performance. To assess the performance of the MaxEnt model, this study employed a multi-metric validation system: an average AUC value of 0.933 was obtained through ROC curve analysis based on 10 replicated runs (Figure 3), and the Kappa coefficient (0.704) and TSS value (0.960) were calculated using the SDMtune package, indicating that the model possesses reliable predictive capability. For identifying key environmental drivers of *A. horsfieldii* distribution, we first performed preliminary screening using Pearson correlation coefficients, followed by variable selection optimization through regularized training gain and Jackknife tests. From 20 candidate variables, we systematically identified seven decisive factors (Table 2): mean diurnal range (bio2), temperature annual range (bio7), precipitation of driest quarter (bio17), altitude (elev), precipitation seasonality (bio15), precipitation of coldest quarter (bio19), and precipitation of driest month (bio14), which collectively accounted for 99.9% of cumulative contribution and 100% permutation importance. Jackknife analysis revealed (Figure 4) that bio2, bio7, bio17, and bio19 showed the most significant gain effects in “only this variable” tests, indicating their substantial individual explanatory power. Conversely, bio15 exhibited the shortest gain bar in “excluding this variable” tests, highlighting its unique environmental information. Our comprehensive analysis identifies bio2, bio7, bio17, and bio19 as the four core environmental determinants shaping the species’ distribution pattern, with two temperature-related and two precipitation-related variables, confirming the dominant role of climatic factors and revealing that *A. horsfieldii’s* response to environmental changes primarily depends on the synergistic effects of temperature fluctuations and extreme seasonal precipitation patterns.

### 3.2. The Relationship Between Each Climate Variable and the Potential Distribution of A. horsfieldii

**Figure 5:**
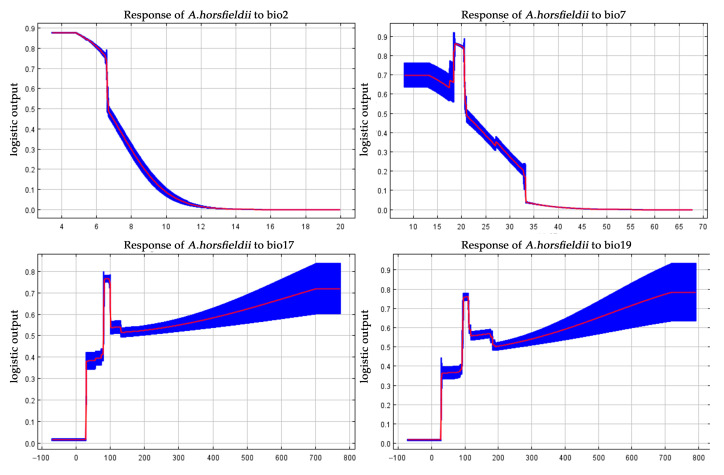
Response curves of major climate variables affecting the distribution of *A. horsfieldii*.

Figure 5 demonstrates that the response curves of climatic factors affecting the survival probability of *A. horsfieldii* exhibit two predominant patterns. For temperature-related variables, the species’ presence probability initially remains stable with increasing climatic index values and then undergoes a gradual decline followed by a rapid decrease, ultimately approaching zero. Specifically, in the response curve for mean diurnal range (bio2), the presence probability peaks at 0.87 within the temperature range of 3.52–4.89 °C. As temperatures rise to 6.53 °C, the probability shows its first significant decrease to 0.75, followed by a sharp drop to 0.49 when exceeding 6.70 °C, with continued gradual decline eventually approaching zero. Similarly, the temperature annual range (bio7) response curve shows the presence probability maintaining a relatively stable high level (0.69) between 8.17 and 13.26 °C. At 17.2 °C, and the probability experiences its first marked decrease to 0.62. However, the curve then displays an anomalous upward trend, reaching a peak (0.86) at 18.52 °C, beyond which the presence probability declines rapidly, plunging to 0.48 at 20.94 °C and gradually approaching zero with further temperature increases.

In contrast to temperature factors, precipitation variables exhibit more complex nonlinear effects on the survival probability of *A. horsfieldii*, characterized by multi-stage fluctuations with alternating increasing and decreasing phases. The response curve for precipitation of driest quarter (bio17) demonstrates this pattern clearly: when precipitation remains at extremely low levels (−71.98 to 27.82 mm), the presence probability stays at a baseline of merely 0.02. As precipitation increases to 27.82 mm, the probability surges rapidly to form the first minor peak (0.38), followed by a gradual rise to 0.41 at 79.85 mm. Subsequent precipitation increases lead to accelerated probability growth, reaching a global maximum (0.76) at 80.35 mm and maintaining this stable level through 80.35–96.11 mm. Beyond 96.11 mm, however, the probability undergoes stepwise declines—plunging sharply to 0.54 at 96.61 mm and persisting at this level until 131.13 mm, then decreasing further to 0.51. Notably, when precipitation exceeds 131.13 mm, the downward trend reverses into a gradual recovery, ultimately stabilizing at a high level of 0.72 beyond 700.19 mm. An analogous multi-phasic response pattern emerges in the precipitation of coldest quarter (bio19) curve: the presence probability remains at a minimal baseline (0.01) under extreme drought conditions (−69.61 to 28.80 mm) and then jumps rapidly to form its first minor peak (0.36) at 29.89 mm and maintains stability through 29.89–75.20 mm. The probability then enters an accelerated growth phase, attaining its global maximum (0.76) at 95.31 mm and sustaining this plateau through 95.31–107.83 mm. When precipitation surpasses 107.83 mm, the probability displays characteristic stepwise decreases—first dropping abruptly to 0.55 at 117.18 mm and then recovering slightly to 0.56 by 174.42 mm, before declining again to 0.50 at 190.65 mm. However, with continued precipitation increases beyond this point, the probability initiates a slow but steady recovery, eventually reaching and maintaining a high stable level of 0.78 when precipitation exceeds 714.34 mm.

### 3.3. The Potential Distribution and Suitability Assessment of A. horsfieldii Under the Current Climate Scenario

**Figure 6:**
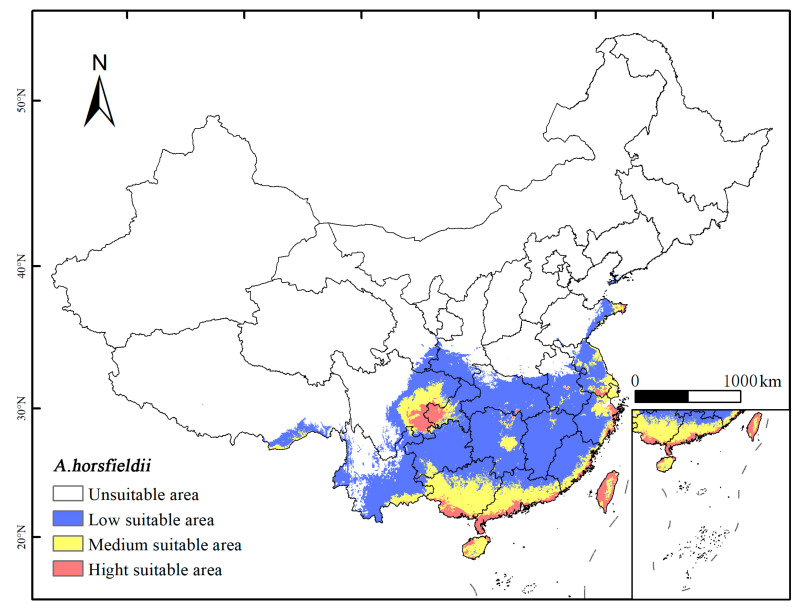
Potential suitable distribution of *A. horsfieldii* under contemporary climate conditions. White, un-suitable habitat area; blue, low-suitability habitat area; orange, moderate-suitability habitat area; pink, high-suitability habitat area.

This study utilized ArcGIS 10.8.0 to reclassify the results of the MaxEnt 3.4.4 model and conducted distribution statistics and area analysis of the different suitable habitats for *A. horsfieldii*. The research indicates (Figure 6) that the total area of potential suitable distribution for *A. horsfieldii* is 212.394 × 10⁴ km^2^, primarily located in central China, south China, east China, southwest China, and northwest China. Among these regions, the highly suitable area covers approximately 15.915 × 10⁴ km^2^, mainly distributed in the southern part of the Guangxi Zhuang Autonomous Region, the southern part of Guangdong Province, the southeastern part of Fujian Province, the eastern part of Zhejiang Province, the southern part of Jiangsu Province, the eastern part of Shandong Province, and Taiwan Province—coastal regions adjacent to the South China Sea and East China Sea, as well as the eastern part of Sichuan Province and the western part of Chongqing Municipality. The moderately suitable area spans about 44.970 × 10⁴ km^2^, primarily surrounding the highly suitable zones, including the eastern part of Sichuan Province, the western part of Chongqing Municipality, the Guangxi Zhuang Autonomous Region, Guangdong Province, most parts of Hainan Province, the southeastern part of Fujian Province, the eastern part of Zhejiang Province, the central and southern parts of Jiangsu Province, the eastern part of Shandong Province, and the central part of Taiwan Province. Additionally, suitable habitats are also found in the eastern part of Hunan Province, the eastern part of Hubei Province, the northern part of Jiangxi Province, the southern part of Anhui Province, the eastern part of Yunnan Province, and the eastern part of the Tibet Autonomous Region. The low-suitability area covers approximately 151.509 × 10⁴ km^2^, mainly distributed in Yunnan Province, the eastern part of Sichuan Province, the eastern part of Chongqing Municipality, Guizhou Province, Hubei Province, Hunan Province, Jiangxi Province, Fujian Province, Anhui Province, Jiangsu Province, and Zhejiang Province, as well as the eastern part of Gansu Province, the southern part of Shaanxi Province, the southern part of Henan Province, the eastern part of Shandong Province, and the eastern part of the Tibet Autonomous Region.

### 3.4. Prediction of the Suitable Habitat of A. horsfieldii Under Future Climate Scenarios

**Figure 7:**
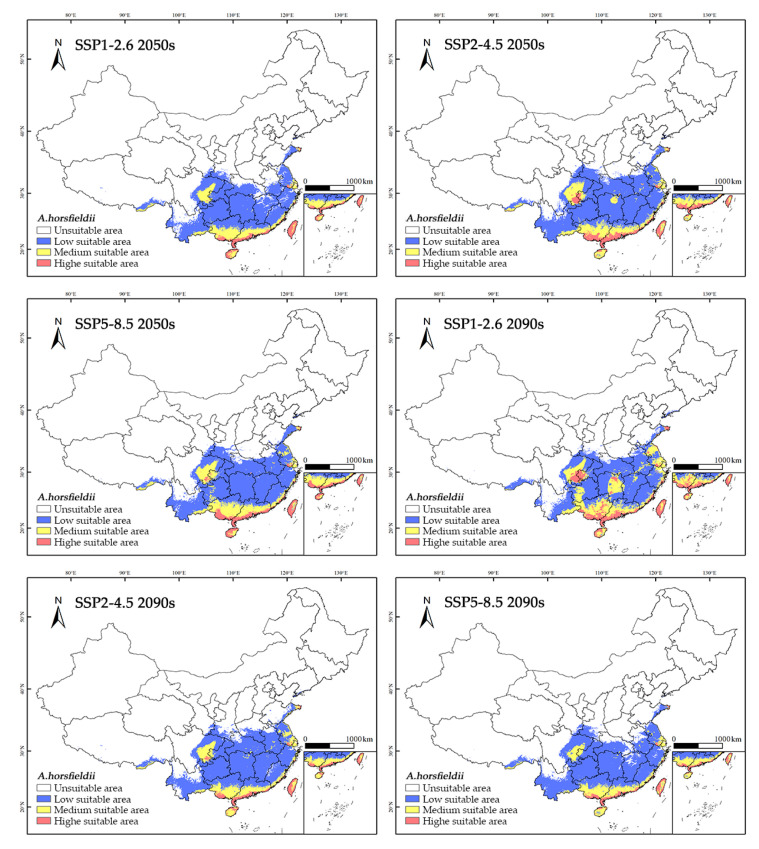
Future species distribution models of *A. horsfieldii* in China under different climate conditions predicated by Maxent. White, un-suitable habitat area; blue, low-suitability habitat area; orange, moderate-suitability habitat area; pink, high-suitability habitat area.

Comparative analysis of the predicted outcomes under six climate scenarios and current potential suitable habitat data (Table 3 and Figure 7) reveals that the future distribution pattern of *A. horsfieldii* will be characterized by “continued contraction of moderately and highly suitable habitats alongside significant expansion of low-suitability areas”. Specifically, under the SSP2-4.5 and SSP5-8.5 scenarios, the low-suitability area demonstrates a steady increasing trend, reaching 166.432 × 10⁴ km^2^ (9.85% increase) and 158.859 × 10⁴ km^2^ (4.85% increase) by the 2050s, respectively. By the 2090s, these areas further expand to 167.411 × 10⁴ km^2^ (10.50% increase) and 159.222 × 10⁴ km^2^ (5.09% increase). Concurrently, the moderately and highly suitable habitats exhibit accelerated decline. The highly suitable area sharply decreases to 9.903 × 10⁴ km^2^ (37.78% reduction) and 8.342 × 10⁴ km^2^ (47.58% reduction) by the 2090s under SSP2-4.5 and SSP5-8.5, respectively. Similarly, the moderately suitable area declines to 30.288 × 10⁴ km^2^ (32.65% reduction) by the 2050s under SSP1-2.6 and to 31.266 × 10⁴ km^2^ (30.47% reduction) by the 2090s under SSP5-8.5. These findings suggest that future climate change may lead to substantial shrinkage of moderately and highly suitable habitats for *A. horsfieldii*, while its distribution range could shift toward low-suitability zones in central, east, and southwest China, including Chongqing Municipality, Henan Province, and Anhui Province.

### 3.5. Analysis of Centroid Migration of A. horsfieldii’s Potential Suitable Habitat in China Under Climate Change Scenarios

**Figure 8:**
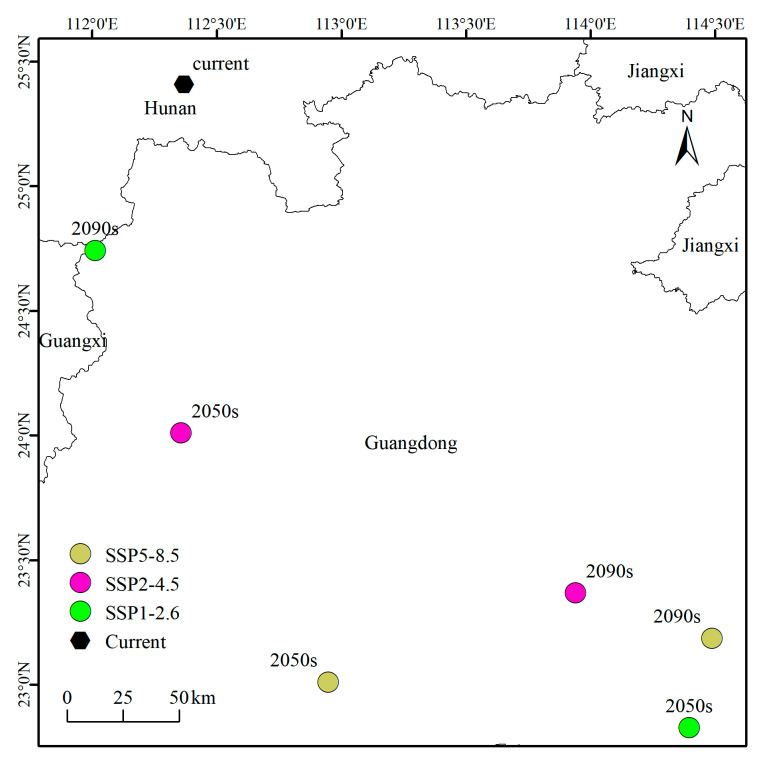
Changes in the centroids of the potential suitability areas for *A. horsfieldii* in China.

The migration trajectory of the centroid for *A. horsfieldii’s* suitable habitats under current and future climate scenarios is shown in Figure 8. Under current climatic conditions, the centroid of *A. horsfieldii* distribution is located in Hunan Province (112.37° E, 25.41° N), exhibiting a distinct southward- and westward-shifting trend. Under the SSP1-2.6 scenario, the centroid migrates from eastern Guangdong (114.40° E, 22.83° N) in the 2050s to northwestern Guangdong (112.01° E, 24.74° N) by the 2090s, forming a southeast-to-northwest migration trajectory. The SSP2-4.5 scenario shows the centroid shifting from western Guangdong (112.36° E, 24.01° N) in the 2050s to a more southwestern position (113.94° E, 23.37° N) in the 2090s. Under the SSP5-8.5 scenario, the centroid moves from eastern Guangdong (112.95° E, 23.01° N) in the 2050s to a southeastern location (114.49° E, 23.19° N) by the 2090s. Comprehensive analysis reveals that under all three climate scenarios, the centroids of *A. horsfieldii*’s suitable habitats demonstrate a tendency to concentrate within Guangdong Province, with an overall migration pattern characterized by shifts from inland to coastal areas and from higher to lower latitudes.

## 4. Discussion

This study utilized the maximum entropy (MaxEnt) model to predict the potential distribution of *A. horsfieldii*. In current research on predicting species’ potential climatic suitability, various ecological niche models including CLIMEX, GARP, GLM, and BIOCLIM have been extensively employed [30,31,32,33]. The MaxEnt model was selected in this study primarily due to its algorithmic characteristics: as a correlative model based on species-occurrence data and environmental background data, it does not rely on the generation of pseudo-absence data. This feature allows MaxEnt to maintain stable modeling performance even when sample sizes are limited [22]. To optimize prediction reliability and accuracy, we systematically tuned model parameters, ultimately identifying the optimal combination (feature type qt, regularization multiplier rm = 2). Model validation yielded excellent performance metrics: AUC = 0.933, Kappa = 0.704, and true skill statistic (TSS) = 0.960, indicating robust predictive capacity for *A. horsfieldii* distribution patterns. The predicted suitable habitats showed strong concordance with actual field occurrence records, accurately reflecting the species’ ecological requirements. Considering the potential correlations between bioclimatic variables, this study combined the Jackknife test and Pearson correlation analysis for variable selection. Additionally, a regularization multiplier (rm = 2) was used to control multicollinearity between variables, effectively optimizing the model structure and further improving the accuracy of the predictive results.

The growing impacts of climate change on ecosystems and species distributions have drawn increasing attention, driven by intensifying international trade, persistent global warming, shifting population densities, land-use transformations, and projected fluctuations in precipitation patterns and intensity [12,34]. Numerous studies and field observations demonstrate that climate change profoundly alters species’ habitat configurations while significantly affecting their reproductive behaviors and distribution ranges [35,36]. For small-bodied organisms like insects, climatic factors play particularly crucial roles in their development and spatial distribution. Under global warming scenarios, rising temperatures and altered precipitation patterns are driving insect population migrations or expansions, potentially leading to species extinctions or biological invasions [37]. Focusing on the potential distribution of *A. horsfieldii*, our study employed Jackknife analysis to identify four key environmental determinants: mean diurnal range (Bio2), temperature annual range (Bio7), precipitation of driest quarter (Bio17), and precipitation of coldest quarter (Bio19). These temperature- and precipitation-related factors directly govern insect development, reproductive cycles, population dynamics, and habitat selection [38]. Insect growth and survival exhibit strong temperature dependence, particularly in regions with elevated or highly variable temperatures that significantly influence life cycles, adaptive capacity, and habitat preferences [39,40]. Precipitation patterns equally critically affect insect habitats through humidity-mediated impacts on food resources, breeding sites, and behavioral ecology [41,42]. Specifically, *A. horsfieldii* displays heightened sensitivity to temperature and precipitation changes, exhibiting a life-history strategy of one generation per year in tropical regions that may extend to two years in cooler climates [43]. (1) Thermal adaptation analysis revealed distinct ecological thresholds: For Bio2, survival probability peaked (0.87) at daily temperature variations of 3.52–4.89 °C, indicating optimal adaptation to stable thermal regimes, while exceeding 6.70 °C caused significant decline to 0.49, suggesting metabolic disruption. Bio7 analysis showed stronger adaptation (0.69 probability) within annual variations of 8.17–13.26 °C, with survival probability dropping to 0.62 at 17.2 °C. Although a temporary rebound occurred at 18.52 °C (0.86), the subsequent rapid decline to 0.48 indicated constrained tolerance to annual thermal fluctuations. Collectively, these patterns demonstrate *A. horsfieldii*’s preference for thermally stable environments with minimal diurnal variation. (2) Hydrological responses exhibited complex multimodal patterns: For Bio17, survival probability remained minimal (0.02) below 27.82 mm but peaked at 80.35 mm (0.76), suggesting optimal host-plant hydration for egg/larval development. Exceeding 96.11 mm precipitated sharp declines, potentially from pathogen proliferation. Bio19 showed parallel trends—near-zero survival (0.01) below 28.80 mm, peaking at 95.31 mm (0.76) for optimal overwintering humidity. While extreme precipitation (714.34 mm) showed gradual stabilization, *A. horsfieldii* likely employs behavioral plasticity and microhabitat selection to buffer hydrological variability. Notably, while these bioclimatic variables effectively capture macro-scale patterns, microhabitat conditions (e.g., canopy and trunk microclimates) often diverge from regional models yet critically determine survival outcomes for small insects [44].

The prediction results of the MaxEnt model indicate that the suitable habitat for *A. horsfieldii* in China is primarily distributed between 90° E and 130° E longitude and between 10° N and 40° N latitude, covering several regions including central China, south China, east China, southwest China, and northwest China. These areas are mainly characterized by subtropical and tropical monsoon climates. Notably, the climatic conditions in these regions are crucial for the growth, reproduction, and habitat selection of *A. horsfieldii*, with variations in temperature and precipitation directly influencing fluctuations in its population size. Looking ahead, the highly and moderately suitable areas for *A. horsfieldii* are expected to shrink significantly; however, its potential distribution is projected to expand into the low-suitability zones of central, eastern, and southwestern China. In particular, regions such as Chongqing, Henan Province, and Anhui Province may become newly suitable habitats. It is worth noting that under three typical climate scenarios (SSP1-2.6, SSP2-4.5, and SSP5-8.5), the centroid of *A. horsfieldii*’s suitable habitat shows a trend of migration concentrating within Guangdong Province. Overall, this exhibits a typical spatial distribution pattern of movement “from inland to coastal regions, and from higher to lower latitudes”. This migration pattern is primarily regulated by a dual climate-driven mechanism: on the one hand, the temperature threshold-exceedance effect leads to frequent extreme summer heat in high-latitude regions, potentially surpassing the upper thermal tolerance limit of *A. horsfieldii*, while rising winter temperatures in lower-latitude areas significantly enhance overwinter survival rates, thereby facilitating a southward shift of suitable habitats [45]; on the other hand, the increased spatiotemporal heterogeneity of precipitation in the Yangtze River Basin—such as alternating seasonal droughts and heavy rainfall—may degrade the quality of oviposition microhabitats [46]. Additionally, changes in precipitation patterns (e.g., decreased rainfall and increased frequency of extreme weather events) may reshape insect distribution patterns through a “habitat fragmentation–rebalancing” mechanism [47], leading to a systematic reorganization of current suitable areas, while new potential habitats may gradually emerge as climate zones shift southward or eastward.

In summary, *Anoplophora horsfieldii* demonstrates extensive habitat suitability potential across China, with its optimal distribution primarily concentrated in the warm, humid climatic regions of central, south, East, and Southwest China that feature favorable precipitation conditions. These areas, characterized by abundant ecological resources and high biodiversity, provide advantageous conditions for its dispersal. Model projections indicate a probable contraction in highly suitable areas alongside an expansion trend in low-suitability zones. Notably, the centroid of *A. horsfieldii*’s suitable habitat exhibits a distinct migration pattern characterized by shifts “from inland to coastal areas and from higher to lower latitudes”. This emerging scenario urgently necessitates the development of effective prevention and control strategies. The implementation of precise monitoring, early warning systems, and targeted management measures will not only effectively contain the spread of *A. horsfieldii* infestations but also provide practical foundations for scientific pest management. Such strategies not only prove valuable for current control operations but also establish crucial theoretical frameworks and practical experience for future ecological conservation and biosecurity efforts. Consequently, we strongly recommend that forestry authorities enhance risk awareness, adopt more efficient control measures, reduce management costs, and prevent further expansion of *A. horsfieldii* into new territories. These proactive approaches are essential for mitigating potential ecological impacts and avoiding significant economic losses associated with this invasive pest.

## 5. Conclusions

This study employed the MaxEnt model and ArcGIS software to predict the current and future (2050s and 2090s) potential distribution of *A. horsfieldii* under six climate scenarios. The results indicate that under current climatic conditions, the total area of potentially suitable habitat for *A. horsfieldii* spans 212.394 × 10⁴ km^2^, primarily distributed across central, south, east, southwest, and northwest China. For the three future climate scenarios (SSP1-2.6, SSP2-4.5, and SSP5-8.5), both highly and moderately suitable areas are projected to significantly decrease, while low-suitability zones may expand further into central, east, and southwest China, with Chongqing, Henan, and Anhui likely emerging as new suitable regions. Notably, the centroid of suitable habitats demonstrates a distinct migratory trend toward Guangdong Province, exhibiting a characteristic distribution shift pattern described as “from inland to coastal areas and from higher to lower latitudes”. The study identified four key limiting factors influencing *A. horsfieldii* distribution: mean diurnal range (Bio2), temperature annual range (Bio7), precipitation of driest quarter (Bio17), and precipitation of coldest quarter (Bio19). These findings provide crucial theoretical guidance for forestry authorities in developing strategies to control the spread of *A. horsfieldii*, while also establishing a solid scientific foundation and practical framework for future ecological conservation and biosecurity efforts.

## Figures and Tables

**Table 1:** Environmental variables of potential geographical distribution of *A. horsfieldii*.

Symbol	Environmental Variable	Unit
Mean diurnal range (mean of monthly) (max temp–min temp)	bio2	°C
Temperature annual range (bio5–bio6)	bio7	°C
Precipitation of driest quarter	bio17	mm
Altitude	elev	m
Precipitation seasonality (coefficient of variation)	bio15	%
Precipitation of coldest quarter	bio19	mm
Precipitation of driest month	bio14	mm

**Table 2:** The contribution rate of climatic factors to the construction of the MaxEnt model.

Variables	Percent Contribution/%	Permutation Importance/%
bio2	41.4	40.2
bio07	28.2	41.9
bio17	23.1	1.2
elev	5.3	10.1
bio15	1.7	6.1
bio19	0.1	0.3
bio14	0.1	0.2

**Table 3:** Prediction of the suitable habitat area for *A. horsfieldii* in China under current and future climate conditions (km^2^).

Period	Current	2050s			2090s		
		SSP1-2.6	SSP2-4.5	SSP5-8.5	SSP1-2.6	SSP2-4.5	SSP5-8.5
Low-suitable areas	151.509 × 10^4^ km^2^	149.347 × 10^4^ km^2^	166.432 × 10^4^ km^2^	158.859 × 10^4^ km^2^	132.231 × 10^4^ km^2^	167.411 × 10^4^ km^2^	159.222 × 10^4^ km^2^
Moderately suitable areas	44.970 × 10^4^ km^2^	30.288 × 10^4^ km^2^	40.175 × 10^4^ km^2^	40.500 × 10^4^ km^2^	52.161 × 10^4^ km^2^	34.502 × 10^4^ km^2^	31.266 × 10^4^ km^2^
High-suitable areas	15.915 × 10^4^ km^2^	10.929 × 10^4^ km^2^	16.630 × 10^4^ km^2^	14.705 × 10^4^ km^2^	21.413 × 10^4^ km^2^	9.903 × 10^4^ km^2^	8.342 × 10^4^ km^2^
Total suitable areas	212.394 × 10^4^ km^2^	190.564 × 10^4^ km^2^	223.238 × 10^4^ km^2^	214.064 × 10^4^ km^2^	205.806 × 10^4^ km^2^	211.816 × 10^4^ km^2^	198.830 × 10^4^ km^2^

## Data Availability

The data supporting the results are available in a public repository at: https://doi.org/10.6084/m9.figshare.28684907.v1.

## References

[1] Zhang B.L., Zhang J., Zhang D., Feng Y., Qiu J., Ye X.J., Wang B.X. (2023). Complete mitochondrial genome of the longicorn Anoplophora horsfieldii Hope (Coleoptera: Cerambycidae). Mitochondrial DNA Part B.

[2] Hoebeke E. (2002). Revision of the Genus Anoplophora (Coleoptera: Cerambycidae).

[3] Lee S., Ko G., Lee Y., Lee S.K., Kim M., Lee M., Kang K., Ming B., Kim D., Lee S. (2024). The third invasive Anoplophora: Citizen science facilitates uncovering massive abundance of non-native *A. horsfieldii* (Coleoptera: Cerambycidae) in South Korea. J. Asia-Pac. Entomol..

[4] Gressitt J.L. (1942). Destructive Long-Horned Beetle Borers at Canton, China.

[5] Gressitt J.L. (1951). Longicorn beetles of China. Longicornia.

[6] Hua L., Nara H., Yu C. (1993). Longicorn Beetles of Hainan & Guangdong.

[7] Chou W.I. (2004). Iconography of Longhorn Beetles in Taiwan.

[8] Tavakilian G.L., Chevillotte H. (2022). Titan: Base de Données Internationales sur les Cerambycidae ou Longicornes.

[9] Lee S., Choi J., Jang H., Choi W., Kwon W., Kim D., Gim J., Park J., Park S., Kim S. (2023). Establishment of non-native *Anoplophora horsfieldii* (Coleoptera: Cerambycidae) in South Korea. J. Integr. Pest. Manag..

[10] Kim M., Lee S.K., Park Y., Kim Y., Lee M., Nam Y. (2025). Climatic suitability and spread potential of Anoplophora horsfieldii (Coleoptera: Cerambycidae), a newly identified non-native insect on Jeju Island, Korea. Sci. Rep..

[11] Hulme P.E. (2021). Unwelcome exchange: International trade as a direct and indirect driver of biological invasions worldwide. One Earth.

[12] Pyšek P., Hulme P.E., Simberloff D., Bacher S., Blackburn T.M., Carlton J.T., Dawson W., Essl F., Foxcroft L.C., Genovesi P. (2020). Scientists’ warning on invasive alien species. Biol. Rev..

[13] Seebens H., Blackburn T.M., Dyer E.E., Genovesi P., Hulme P.E., Jeschke J.M., Pagad S., Pyšek P., Winter M., Arianoutsou M. (2017). No saturation in the accumulation of alien species worldwide. Nat. Commun..

[14] Renault D., Laparie M., McCauley S.J., Bonte D. (2018). Environmental adaptations, ecological filtering, and dispersal central to insect invasions. Annu. Rev. Entomol..

[15] Poland T.M., Patel-Weynand T., Finch D.M., Miniat C.F., Hayes D.C., Lopez V.M. (2021). Invasive Species in Forests and Rangelands of the United States.

[16] Cao R., Feng J. (2024). Future Climate Change and Anthropogenic Disturbance Promote the Invasions of the World’s Worst Invasive Insect Pests. Insects.

[17] Pecl G.T., Araújo M.B., Bell J.D., Blanchard J., Bonebrake T.C., Chen I.-C., Clark T.D., Colwell R.K., Danielsen F., Evengård B. (2017). Biodiversity redistribution under climate change: Impacts on ecosystems and human well-being. Science.

[18] Liu C., Yang M., Li M., Jin Z., Yang N., Yu H., Liu W. (2024). Climate Change Facilitates the Potentially Suitable Habitats of the Invasive Crop Insect *Ectomyelois ceratoniae* (Zeller). Atmosphere.

[19] Silva D.P., Andrade A.F.A., Oliveira J.P.J., Morais D.M., Vieira J.E.A., Engel M.S. (2019). Current and future ranges of an elusive North American insect using species distribution models. J. Insect Conserv..

[20] Wen X., Fang G., Chai S., He C., Sun S., Zhao G., Lin X. (2024). Can ecological niche models be used to accurately predict the distribution of invasive insects? A case study of Hyphantria cunea in China. Ecol. Evol..

[21] Zhu G., Liu G., Bu W., Gao Y. (2013). The basic principles of niche models and their applications in biodiversity conservation biological diversity. Biodivers. Sci..

[22] Phillips S.J., Anderson R.P., Schapire R.E. (2006). Maximum entropy modeling of species geographic distributions. Ecol. Model..

[23] Alramadan Y., Mamay M., Farooq S. (2025). Increased spread risk of citrus long-horned beetle [*Anoplophora chinensis* (Coleoptera: Cerambycidae)] under climate change in Türkiye: Implications for management. Crop Prot..

[24] Zhang Q., Wang J., Lei Y. (2022). Predicting Distribution of the Asian Longhorned Beetle, Anoplophora glabripennis (Coleoptera: Cerambycidae) and Its Natural Enemies in China. Insects.

[25] Xin X., Wu T., Zhang J., Zhang F., Li W., Zhang Y., Lu Y., Fang Y., Jie W., Zhang L. (2019). Introduction to BCC Mode and CMIP6 Test Conducted. Adv. Clim. Change Res..

[26] Kim T., Cho Y. (2024). Predicting the potential distribution of an invasive species, *Anoplophora horsfieldii* (Coleoptera: Cerambycidae), under climate change using species distribution model. Proc. Korean Soc. Appl. Entomol. Conf..

[27] Cobos M.E., Peterson A.T., Barve N., Osorio-Olvera L. (2019). kuenm: An R package for detailed development of ecological niche models using Maxent. Peerj.

[28] Wang Y., Xie B., Wan F., Xiao Q., Dai L. (2007). Application of ROC curve analysis in evaluating the performance of alien species’ potential distribution models. Biodivers. Sci..

[29] Qin Z., Zhang J.E., DiTommaso A., Wang R.L., Wu R.S. (2015). Predicting invasions of *Wedelia trilobata* (L.) Hitchc. with Maxent and GARP models. J. Plant Res..

[30] Poutsma J., Loomans A.J.M., Aukema B., Heijerman T. (2008). Predicting the potential geographical distribution of the harlequin ladybird, Harmonia axyridis, using the CLIMEX model. From Biological Control to Invasion: The Ladybird Harmonia Axyridis as a Model Species.

[31] Wang G., Zhang H., Cao X., Zhang X., Wang G., He Z., Yu C., Zhao T. (2014). Using GARP to predict the range of Aedes aegypti in China. Southeast Asian J. Trop. Med..

[32] Guisan A., Weiss S.B., Weiss A.D. (1999). GLM versus CCA spatial modeling of plant species distribution. Plant Ecol..

[33] Howse M.W., Haywood J., Lester P.J. (2020). Bioclimatic modelling identifies suitable habitat for the establishment of the invasive European paper wasp (Hymenoptera: Vespidae) across the southern hemisphere. Insects.

[34] Xu Y., Xue F., Lin Y. (2003). Simulation analysis of ground temperature and precipitation changes in China in the 21st century under different greenhouse gas emission scenarios. Clim. Environ. Res..

[35] Roth T., Plattner M., Amrhein V. (2014). Plants, birds and butterflies: Short-term responses of species communities to climate warming vary by taxon and with altitude. PLoS ONE.

[36] Ziter C., Robinson E.A., Newman J.A. (2012). Climate change and voltinism in C alifornian insect pest species: Sensitivity to location, scenario and climate model choice. Global Change Biol..

[37] Skendžić S., Zovko M., Pajač Živković I., Lešić V., Lemić D. (2021). Effect of climate change on introduced and native agricultural invasive insect pests in Europe. Insects.

[38] Kingsolver J.G., Arthur Woods H., Buckley L.B., Potter K.A., MacLean H.J., Higgins J.K. (2011). Complex Life Cycles and the Responses of Insects to Climate Change.

[39] Xing K., Hoffmann A.A., Ma C. (2014). Does thermal variability experienced at the egg stage influence life history traits across life cycle stages in a small invertebrate?. PLoS ONE.

[40] Colinet H., Sinclair B.J., Vernon P., Renault D. (2015). Insects in fluctuating thermal environments. Annu. Rev. Entomol..

[41] Pareek A., Meena B.M., Sharma S., Tetarwal M.L., Kalyan R.K., Meena B.L. (2017). Impact of climate change on insect pests and their management strategies. Clim. Chang. Sustain. Agric..

[42] Liu Y., Luo J., Zhang D., Wei Y., Zhou Z. (2019). The effect of temperature and humidity on the developmental duration and hatching rate of Huangqi root nodule weevil eggs. Acta Phytophylacica Sin..

[43] Duffy E.A.J. (1968). A monograph of the Immature Stages of Oriental Timber Beetles (Cerambycidae).

[44] Potter K.A., Arthur Woods H., Pincebourde S. (2013). Microclimatic challenges in global change biology. Glob. Change Biol..

[45] Raza M.M., Khan M.A., Arshad M., Sagheer M., Sattar Z., Shafi J., Haq E.U., Ali A., Aslam U., Mushtaq A. (2015). Impact of global warming on insects. Arch. Phytopathol. Plant Prot..

[46] Chen H., Sun J. (2015). Assessing model performance of climate extremes in China: An intercomparison between CMIP5 and CMIP3. Clim. Change.

[47] Zhao Z., Luo Y., Jiang Y., Xu Y. (2008). Detection and evaluation of global and Chinese precipitation, drought and flood changes. Sci. Technol. Rev..

